# Subject Index Volume 5

**DOI:** 10.1111/jdi.12293

**Published:** 2014-10-27

**Table:** 

acute coronary syndrome
admission hyperglycemia predicts poorer short- and long-term outcomes after primary percutaneous coronary intervention for ST-elevation myocardial infarction, Chen 80–86
adipocytokines
association between serum adipocytokine levels and microangiopathies in patients with type 2 diabetes mellitus, Jung 333–339
adiponectin
adiponectin gene variants, adiponectin isoforms and cardiometabolic risk in type 2 diabetic patients, Foucan 192–198
adipose tissue
injection of *Lactobacillus casei* strain Shirota affects autonomic nerve activities in a tissue-specific manner, and regulates glucose and lipid metabolism in rats, Tanida 153–161
admission glucose
admission hyperglycemia predicts poorer short- and long-term outcomes after primary percutaneous coronary intervention for ST-elevation myocardial infarction, Chen 80–86
advanced glycation end-products
combined effects of physiologically relevant disturbed wall shear stress and glycated albumin on endothelial cell functions associated with inflammation, thrombosis and cytoskeletal dynamics, Maria 372–381
aerobic exercise
mild-to-moderate intensity exercise improves cardiac autonomic drive in type 2 diabetes, Goit 722–727
afferent vagal nerve
injection of *Lactobacillus casei* strain Shirota affects autonomic nerve activities in a tissue-specific manner, and regulates glucose and lipid metabolism in rats, Tanida 153–161
aging
age-associated changes of islet endocrine cells and the effects of body mass index in Japanese, Mizukami 38–47
albuminuria
association of serum bilirubin levels with development and progression of albuminuria, and decline in estimated glomerular filtration rate in patients with type 2 diabetes mellitus, Toya 228–235
sitagliptin improves albuminuria in patients with type 2 diabetes mellitus, Mori 313–319
albuminuria: prevalence, associated risk factors and relationship with cardiovascular disease, Chen 464–471
severity of hearing impairment is positively associated with urine albumin excretion rate in patients with type 2 diabetes, Shen 743–747
angiotensin II receptor blocker
combination of a peroxisome proliferator-activated receptor-gamma agonist and an angiotensin II receptor blocker attenuates myocardial fibrosis and dysfunction in type 2 diabetic rats, Shim 362–371
anti-oxidants
diabetic cardiomyopathy and its mechanisms: role of oxidative stress and damage, (Review Article) Liu 623–634
anti-oxidant genes
number of circulating pro-angiogenic cells, growth factor and anti-oxidative gene profiles might be altered in type 2 diabetes with and without diabetic foot syndrome, Nowak 99–107
antidiabetic drugs
cancer biology in diabetes (Review Article), Sen 251–264
apolipoprotein M
modulation of sphingosine-1-phosphate and apolipoprotein M levels in the plasma, liver and kidneys in streptozotocin-induced diabetic mice, Nojiri 639–648
arachidonic acid
comparison of the efficacy of sitagliptin and glimepiride dose-up in Japanese patients with type 2 diabetes poorly controlled by sitagliptin and glimepiride in combination, Shimoda 320–326
Asia
dietary intake in Japanese patients with type 2 diabetes: analysis from Japan Diabetes Complications Study, Horikawa 176–187
atherosclerosis
comparison of brachial artery flow-mediated dilation in youth with type 1 and type 2 diabetes mellitus, Ohsugi 615–620
Comparison of carotid and lower limb atherosclerotic lesions in both previously known and newly diagnosed type 2 diabetes mellitus, Li 734–742
autonomic imbalance
association of hypertension status and cardiovascular risks with sympathovagal imbalance in first degree relatives of type 2 diabetics, Pal 449–455
bariatric surgery
effects of bariatric surgery on metabolic and nutritional parameters in severely obese Korean patients with type 2 diabetes: a prospective 2-year follow up, Kim 221–227
basal insulin dose
basal insulin requirement of youth with type 1 diabetes differs according to age, Urakami 442–444
bilirubin
association of serum bilirubin levels with development and progression of albuminuria, and decline in estimated glomerular filtration rate in patients with type 2 diabetes mellitus, Toya 228–235
blood glucose
factors influencing the durability of the glucose-lowering effect of sitagliptin combined with a sulfonylurea, Kubota 445–448
bodyweight
comparison of three α-glucosidase inhibitors for glycemic control and bodyweight reduction in Japanese patients with obese type 2 diabetes, Sugihara 206–212
caloric restriction
weight loss on a structured hypocaloric diet with or without exercise improves emotional distress and quality of life in overweight and obese patients with type 2 diabetes, Wycherley 94–98
cancer
cancer biology in diabetes (Review Article), Sen 251–264
carbohydrate antigen 19–9
decreased serum CA19-9 is associated with improvement of insulin resistance and metabolic control in patients with obesity and type 2 diabetes after Roux-en-Y gastric bypass, Tu 694–700
carbohydrate insulin ratio
diurnal variation of carbohydrate insulin ratio in adult type 1 diabetic patients treated with continuous subcutaneous insulin infusion, Nakamura 48–50
cardiometabolic risk
adiponectin gene variants, adiponectin isoforms and cardiometabolic risk in type 2 diabetic patients, Foucan 192–198
cardiovascular disease
combined effects of physiologically relevant disturbed wall shear stress and glycated albumin on endothelial cell functions associated with inflammation, thrombosis and cytoskeletal dynamics, Maria 372–381
albuminuria: prevalence, associated risk factors and relationship with cardiovascular disease, Chen 464–471
cardiovascular diseases
carotid ultrasonography: a potent tool for better clinical practice in diagnosis of atherosclerosis in diabetic patients (Review Article), Katakami 3–13
comparison of four cardiovascular risk prediction functions among Chinese patients with diabetes mellitus in the primary care setting, Jiao 606–614
cardiovascular risk
association of hypertension status and cardiovascular risks with sympathovagal imbalance in first degree relatives of type 2 diabetics, Pal 449–455
carotid ultrasound
carotid ultrasonography: a potent tool for better clinical practice in diagnosis of atherosclerosis in diabetic patients (Review Article), Katakami 3–13
carpal tunnel syndrome
median neuropathy at the wrist as an early manifestation of diabetic neuropathy, Horinouchi 709–713
β-cell function
secretory units of islets in transplantation index is a useful predictor of insulin requirement in Japanese type 2 diabetic patients, Iwata 570–580
β-cell turnover
age-associated changes of islet endocrine cells and the effects of body mass index in Japanese, Mizukami 38–47
chemerin
pioglitazone and metformin are equally effective in reduction of chemerin in patients with type 2 diabetes, Esteghamati 327–332
circadian hyper-amplitude-tension
variations in 7-day/24-h circadian pattern of ambulatory blood pressure and heart rate of type 2 diabetes patients, Bhardwaj 728–733
cognitive impairment
effect of renal impairment on cognitive function during a 3-year follow up in elderly patients with type 2 diabetes: association with microinflammation, Kawamura 597–605
colorectal adenoma
fasting serum insulin levels and insulin resistance are associated with colorectal adenoma in Koreans, Kim 297–304
combination therapy
effect of combination therapy with repaglinide and metformin hydrochloride on glycemic control in Japanese patients with type 2 diabetes mellitus, Kawamori 72–79
Commentary
adaptation of the brain to hypoglycemia: does the brain stand by for the next hypoglycemia event?, Fukui 284–285
advances around technologies investigating mitochondrial function and insights gained by their applications, Lee 144–146
commentary on the United Kingdom Prospective Diabetes Study outcomes model 2: need for long-term follow up and quality of life data in Asian patients, Tanaka 281–283
dual protein kinase C alpha and beta inhibitors and diabetic kidney disease: a revisited therapeutic target for future clinical trials, Koya 147–148
effect of diabetes on stroke symptoms and mortality: lessons from a recent large population-based cohort study, Umemura 14–16
from sugar to kidney: a never-ending battle, Lin 482–483
glycated hemoglobin variability: a potential new risk marker for diabetes complications?, Jia 635–636
glycated hemoglobin variability and retinopathy progression in type 1 diabetes: is month-to-moth instability a better predictor?, Chang 149–152
improved prediction of cardiovascular events in diabetic patients: role of quantitative ultrasonic tissue characterization, Sheen 17–18
is obesity an absolute evil? increase in adipose tissue does not always decrease insulin sensitivity, Ishikawa 278–280
long-term effect of dipeptidyl peptidase-4 inhibition on β-cell mass in an advanced-aged diet-induced obesity mouse model, Yamane 142–143
look Action for Health in Diabetes trial: what we have learned in terms of real world practice and clinical trials, Kwok 637–638
new liver–β-cell axis that controls insulin secretory capacity, Matsuzaka 276–277
selective and sequential loss of transcriptional factors: a hallmark of β-cell failure in type 2 diabetes?, Shirakawa 359–361
continuous subcutaneous insulin infusion
diurnal variation of carbohydrate insulin ratio in adult type 1 diabetic patients treated with continuous subcutaneous insulin infusion, Nakamura 48–50
C-peptide
postprandial C-peptide to glucose ratio as a predictor of β-cell function and its usefulness for staged management of type 2 diabetes, Lee 517–524
tolerability, effectiveness and predictive parameters for the therapeutic usefulness of exenatide in obese, Korean patients with type 2 diabetes, Song 554–562
criteria
diagnostic criteria for acute-onset type 1 diabetes mellitus (2012): report of the Committee of Japan Diabetes Society on the Research of Fulminant and Acute-onset Type 1 Diabetes Mellitus, Kawasaki 115–118
diabetes
admission hyperglycemia predicts poorer short- and long-term outcomes after primary percutaneous coronary intervention for ST-elevation myocardial infarction, Chen 80–86
one size does not fit all glycemic targets for type 2 diabetes (Review Article), Pozzilli 134–141
adiponectin gene variants, adiponectin isoforms and cardiometabolic risk in type 2 diabetic patients, Foucan 192–198
cancer biology in diabetes (Review Article), Sen 251–264
risk of hospitalization for diabetic macrovascular complications and in-hospital mortality with irregular physician visits using propensity score matching, Nishi 428–434
comparative study on cardiac autonomic modulation during deep breathing test and diaphragmatic breathing in type 2 diabetes and healthy subjects, Subbalakshmi 456–463
epidemic trend of diabetes in China (Review Article), Chen 478–481
prevalence and clinical profile of diabetes mellitus in productive aged urban Indonesians, Mihardja 507–512
factors associated with good glycemic control among patients with type 2 diabetes mellitus, Ahmad 563–569
application of a health-related quality of life conceptual model in community-dwelling older Chinese people with diabetes to understand the relationships among clinical and psychological outcomes, Shiu 677–686
variations in 7-day/24-h circadian pattern of ambulatory blood pressure and heart rate of type 2 diabetes patients, Bhardwaj 728–733
comparison of carotid and lower limb atherosclerotic lesions in both previously known and newly diagnosed type 2 diabetes mellitus, Li 734–742
See also type 1 diabetes
See also type 2 diabetes
diabetes complications
comparison of four cardiovascular risk prediction functions among Chinese patients with diabetes mellitus in the primary care setting, Jiao 606–614
diabetes mellitus
carotid ultrasonography: a potent tool for better clinical practice in diagnosis of atherosclerosis in diabetic patients (Review Article), Katakami 3–13
hepatitis B virus-associated nephropathy in a patient with diabetes mellitus, Zou 87–89
albuminuria: prevalence, associated risk factors and relationship with cardiovascular disease, Chen 464–471
comparison of brachial artery flow-mediated dilation in youth with type 1 and type 2 diabetes mellitus, Ohsugi 615–620
increasing risk of diabetes mellitus according to liver function alterations in electronic workers, Lee 671–676
diabetes mellitus type 2
prevalences of diabetic retinopathy and nephropathy are lower in Korean type 2 diabetic patients with non-alcoholic fatty liver disease, Kim 170–175
diabetes risk
oral glucose tolerance test-based calculation identifies different glucose intolerance phenotypes within the impaired fasting glucose range, Schianca 533–538
diabetic angiopathies
prevalences of diabetic retinopathy and nephropathy are lower in Korean type 2 diabetic patients with non-alcoholic fatty liver disease, Kim 170–175
diabetic cardiomyopathy
combination of a peroxisome proliferator-activated receptor-gamma agonist and an angiotensin II receptor blocker attenuates myocardial fibrosis and dysfunction in type 2 diabetic rats, Shim 362–371
diabetic cardiomyopathy and its mechanisms: role of oxidative stress and damage (Review Article), Liu 623–634
diabetic foot
macrophage stimulating agent soluble yeast β-1,3/1,6-glucan as a topical treatment of diabetic foot and leg ulcers: a randomized, double blind, placebo-controlled phase II study, Zykova 392–399
diabetic foot syndrome
number of circulating pro-angiogenic cells, growth factor and anti-oxidative gene profiles might be altered in type 2 diabetes with and without diabetic foot syndrome, Nowak 99–107
diabetic kidney disease
obesity as an effect modifier of the association between leptin and diabetic kidney disease, Hanai 213–220
diabetic macrovascular complications
risk of hospitalization for diabetic macrovascular complications and in-hospital mortality with irregular physician visits using propensity score matching, Nishi 428–434
diabetic microangiopathies
association between serum adipocytokine levels and microangiopathies in patients with type 2 diabetes mellitus, Jung 333–339
diabetic nephropathy
hepatitis B virus-associated nephropathy in a patient with diabetes mellitus, Zou 87–89
association of serum bilirubin levels with development and progression of albuminuria, and decline in estimated glomerular filtration rate in patients with type 2 diabetes mellitus, Toya 228–235
diabetic neuropathy
median neuropathy at the wrist as an early manifestation of diabetic neuropathy, Horinouchi 709–713
diabetic peripheral neuropathy
assessment of vibratory sensation with a tuning fork at different sites in Japanese patients with diabetes mellitus, Takahara 90–93
prevalence and risk factors of development of peripheral diabetic neuropathy in type 2 diabetes mellitus in a tertiary care setting, Bansal 714–721
diagnosis
diagnostic criteria for acute-onset type 1 diabetes mellitus (2012): report of the Committee of Japan Diabetes Society on the Research of Fulminant and Acute-onset Type 1 Diabetes Mellitus, Kawasaki 115–118
diaphragmatic
comparative study on cardiac autonomic modulation during deep breathing test and diaphragmatic breathing in type 2 diabetes and healthy subjects, Subbalakshmi 456–463
dietary intake
appropriate physical activity and dietary intake achieve optimal metabolic control in older type 2 diabetes patients, Huang 418–427
diethylnitrosamine
role of insulin receptor substrate-1 for diethylnitrosamine plus high-fat diet-induced hepatic tumorigenesis in mice, Zolzaya 27–30
dipeptidyl peptidase-4 inhibitor
efficacy and safety of nateglinide plus vildagliptin combination therapy compared with switching to vildagliptin in type 2 diabetes patients inadequately controlled with nateglinide, Kudo-Fujimaki 400–409
sitagliptin improves albuminuria in patients with type 2 diabetes mellitus, Mori 313–319
short- and long-term effect of sitagliptin after near normalization of glycemic control with insulin in poorly controlled Japanese type 2 diabetic patients, Fujisawa 548–553
durability
predictive factors of durability to sitagliptin: slower reduction of glycated hemoglobin, older age and higher baseline glycated hemoglobin, Chung 51–59
Editorial
are both protein kinase A- and protein kinase C-dependent pathways involved in glucagon-like peptide-1 action on pancreatic insulin secretion?, Shigeto 347–348
dipeptidyl peptidase-4 inhibitors and sulfonylureas for type 2 diabetes: friend or foe?, Yabe 475–477
diabetes and periodontal disease: what should we learn next?, Naruse 249–250
epigenetics: reprogrammable interface of the genome and environments, Sasaki 119–120
success of 2013–2020 World Health Organization action plan to control non-communicable diseases would require pollutants control, Lee 621–622
we have become an Open Access journal!, Hotta 1–2
elderly
one-min mental status examination for category fluency is more useful than mini-mental state examination to evaluate the reliability of insulin self-injection in elderly diabetic patients, Yajima 340–344
epidemiology
epidemic trend of diabetes in China (Review Article), Chen 478–481
comparison of carotid and lower limb atherosclerotic lesions in both previously known and newly diagnosed type 2
diabetes mellitus, Li 734–742
endurance training
effect of endurance training on retinol-binding protein 4 gene expression and its protein level in adipose tissue and the liver in diabetic rats induced by a high-fat diet and streptozotocin, Mansouri 484–491
exenatide
tolerability, effectiveness and predictive parameters for the therapeutic usefulness of exenatide in obese, Korean patients with type 2 diabetes, Song 554–562
fasting plasma glucose
relationship and factors responsible for regulating fasting and post-challenge plasma glucose levels in the early stage development of type 2 diabetes mellitus, Aoyama-Sasabe 663–670
fasting sugar
role of peroxisome proliferator-activated receptor gamma gene polymorphisms in type 2 diabetes mellitus patients of West Bengal, India, Pattanayak 188–191
first-degree relatives of type 2 diabetics
association of hypertension status and cardiovascular risks with sympathovagal imbalance in first degree relatives of type 2 diabetics, Pal 449–455
food intake
dietary intake in Japanese patients with type 2 diabetes: analysis from Japan Diabetes Complications Study, Horikawa 176–187
fracture
fracture is additionally attributed to hyperhomocysteinemia in men and premenopausal women with type 2 diabetes, Li 236–241
gastric inhibitory polypeptide
sensory and motor physiological functions are impaired in gastric inhibitory polypeptide receptor-deficient mice, Okawa 31–37
gender
hemoglobin A1c values are affected by hemoglobin level and gender in non-anemic Koreans, Bae 60–65
Genome-wide Gene-Based Association Study
identification of novel risk genes associated with type 1 diabetes mellitus using a genome-wide gene-based association analysis, Qiu 649–656
gingival/periodontal inflammation
potential association between prediabetic conditions and gingival and/or periodontal inflammation, Andriankaja 108–114
glimepiride
comparison of the efficacy of sitagliptin and glimepiride dose-up in Japanese patients with type 2 diabetes poorly controlled by sitagliptin and glimepiride in combination, Shimoda 320–326
glimepiride/metformin combination
efficacy of glimepiride/metformin fixed-dose combination vs metformin uptitration in type 2 diabetic patients inadequately controlled on low-dose metformin monotherapy: a randomized, open label, parallel group, multicenter study in Korea, Kim 701–708
glinides
efficacy and safety of sitagliptin in Japanese patients with type 2 diabetes switched from glinides, Tanaka 199–205
efficacy and safety of nateglinide plus vildagliptin combination therapy compared with switching to vildagliptin in type 2 diabetes patients inadequately controlled with nateglinide, Kudo-Fujimaki 400–409
β-glucans
macrophage stimulating agent soluble yeast β-1,3/1,6-glucan as a topical treatment of diabetic foot and leg ulcers: a randomized, double blind, placebo-controlled phase II study, Zykova 392–399
glucose control
one size does not fit all glycemic targets for type 2 diabetes (Review Article), Pozzilli 134–141
α-glucoseidase inhibitor
comparison of three α-glucosidase inhibitors for glycemic control and bodyweight reduction in Japanese patients with obese type 2 diabetes, Sugihara 206–212
glycemic control
factors associated with good glycemic control among patients with type 2 diabetes mellitus, Ahmad 563–569
changes in oral antidiabetic prescriptions and improved glycemic control during the years 2002–2011 in Japan (JDDM32), Oishi 581–587
heart rate variability
mild-to-moderate intensity exercise improves cardiac autonomic drive in type 2 diabetes, Goit 722–727
hemoglobin A1c
hemoglobin A1c values are affected by hemoglobin level and gender in non-anemic Koreans, Bae 60–65
factors associated with good glycemic control among patients with type 2 diabetes mellitus, Ahmad 563–569
hepatic tumorigenesis
role of insulin receptor substrate-1 for diethylnitrosamine plus high-fat diet-induced hepatic tumorigenesis in mice, Zolzaya 27–30
hepatitis B virus-associated nephropathy
hepatitis B virus-associated nephropathy in a patient with diabetes mellitus, Zou 87–89
hepatocyte nuclear factor 1α
low serum level of high-sensitivity C-reactive protein in a Japanese patient with maturity-onset diabetes of the young type 3 (MODY3), Ohki 513–516
high-density lipoprotein cholesterol
low high-density lipoprotein cholesterol level is a significant risk factor for development of type 2 diabetes: data from the Hawaii–Los Angeles–Hiroshima study, Hirano 501–506
high-fat diet
modeling type 2 diabetes in rats using high fat diet and streptozotocin (Review Article), Skovsø 349–358
high-sensitivity C-reactive protein
low serum level of high-sensitivity C-reactive protein in a Japanese patient with maturity-onset diabetes of the young type 3 (MODY3), Ohki 513–516
homocysteine
fracture is additionally attributed to hyperhomocysteinemia in men and premenopausal women with type 2 diabetes, Li 236–241
hormone
interactive effects of an isocaloric high-protein diet and resistance exercise on body composition, ghrelin, and metabolic and hormonal parameters in untrained young men: a randomized clinical trial, Kim 242–247
hyperglycemia
injection of *Lactobacillus casei* strain Shirota affects autonomic nerve activities in a tissue-specific manner, and regulates glucose and lipid metabolism in rats, Tanida 153–161
randomized, placebo-controlled, double-blind glycemic control trial of novel sodium-dependent glucose cotransporter 2 inhibitor ipragliflozin in Japanese patients with type 2 diabetes mellitus, Kashiwagi 382–391
hyperglycemic crisis
clinical characteristics of hyperglycemic crises in patients without a history of diabetes, Chou 657–662
impaired fasting glucose
oral glucose tolerance test-based calculation identifies different glucose intolerance phenotypes within the impaired fasting glucose range, Schianca 533–538
impaired glucose
potential association between prediabetic conditions and gingival and/or periodontal inflammation, Andriankaja 108–114
incretins
sensory and motor physiological functions are impaired in gastric inhibitory polypeptide receptor-deficient mice, Okawa 31–37
insulin
fasting serum insulin levels and insulin resistance are associated with colorectal adenoma in Koreans, Kim 297–304
one-min mental status examination for category fluency is more useful than mini-mental state examination to evaluate the reliability of insulin self-injection in elderly diabetic patients, Yajima 340–344
insulin regimens
evaluation of insulin regimens as an effective option for glycemic control in patients with type 2 diabetes: a propensity score-matched cohort study across Japan (JDDM31), Kanatsuka 539–547
secretory units of islets in transplantation index is a useful predictor of insulin requirement in Japanese type 2 diabetic patients, Iwata 570–580
insulin resistance
fasting serum insulin levels and insulin resistance are associated with colorectal adenoma in Koreans, Kim 297–304
usefulness of the insulin tolerance test in patients with type 2 diabetes receiving insulin therapy, Okita 305–312
effect of endurance training on retinol-binding protein 4 gene expression and its protein level in adipose tissue and the liver in diabetic rats induced by a high-fat diet and streptozotocin, Mansouri 484–491
decreased serum CA19-9 is associated with improvement of insulin resistance and metabolic control in patients with obesity and type 2 diabetes after Roux-en-Y gastric bypass, Tu 694–700
insulin secretion
efficacy and safety of nateglinide plus vildagliptin combination therapy compared with switching to vildagliptin in type 2 diabetes patients inadequately controlled with nateglinide, Kudo-Fujimaki 400–409
insulin therapy
usefulness of the insulin tolerance test in patients with type 2 diabetes receiving insulin therapy, Okita 305–312
short- and long-term effect of sitagliptin after near normalization of glycemic control with insulin in poorly controlled Japanese type 2 diabetic patients, Fujisawa 548–553
insulin tolerance test
usefulness of the insulin tolerance test in patients with type 2 diabetes receiving insulin therapy, Okita 305–312
intramuscular fat
effects of bariatric surgery on metabolic and nutritional parameters in severely obese Korean patients with type 2 diabetes: a prospective 2-year follow up, Kim 221–227
irregular visits
risk of hospitalization for diabetic macrovascular complications and in-hospital mortality with irregular physician visits using propensity score matching, Nishi 428–434
islet structure
age-associated changes of islet endocrine cells and the effects of body mass index in Japanese, Mizukami 38–47
insulin receptor substrate-1
role of insulin receptor substrate-1 for diethylnitrosamine plus high-fat diet-induced hepatic tumorigenesis in mice, Zolzaya 27–30
insulin resistance
accurate method to estimate insulin resistance from multiple regression models using data of metabolic syndrome and oral glucose tolerance test, Wu 290–296
knowledge-based mining system for Genome-wide Genetic studies
identification of novel risk genes associated with type 1 diabetes mellitus using a genome-wide gene-based association analysis, Qiu 649–656
Korea
efficacy of glimepiride/metformin fixed-dose combination vs metformin uptitration in type 2 diabetic patients inadequately controlled on low-dose metformin monotherapy: a randomized, open label, parallel group, multicenter study in Korea, Kim 701–708
leptin
obesity as an effect modifier of the association between leptin and diabetic kidney disease, Hanai 213–220
Letter to the Editor
decreased expression of platelet human scavenger receptor class B type I in patients with type 2 diabetes mellitus, Imachi 345–346
case of fulminant type 1 diabetes mellitus associated with parvovirus B19 infection, Nishiumi 472–473
case of fulminant type 1 diabetes with coronary microcirculatory dysfunction, Yamada 748–749
lifestyle intervention
weight loss on a structured hypocaloric diet with or without exercise improves emotional distress and quality of life in overweight and obese patients with type 2 diabetes, Wycherley 94–98
lipotoxicity
palmitate induces reactive oxygen species production and β-cell dysfunction by activating nicotinamide adenine dinucleotide phosphate oxidase through Src signaling, Sato 19–26
liraglutide
oral glucose-stimulated serum C-peptide predicts successful switching from insulin therapy to liraglutide monotherapy in Japanese patients with type 2 diabetes and renal impairment, Araki 435–441
liver function
increasing risk of diabetes mellitus according to liver function alterations in electronic workers, Lee 671–676
low-income outpatients
reward-based, task-setting education strategy on glycemic control and self-management for low-income outpatients with type 2 diabetes, Guo 410–417
maturity-onset diabetes of the young type 3
low serum level of high-sensitivity C-reactive protein in a Japanese patient with maturity-onset diabetes of the young type 3 (MODY3), Ohki 513–516
mean platelet volume
correlation between mean platelet volume and blood glucose levels after oral glucose loading in normoglycemic and prediabetic Japanese subjects, Shimodaira 66–71
medial malleolus
assessment of vibratory sensation with a tuning fork at different sites in Japanese patients with diabetes mellitus, Takahara 90–93
median neuropathy
median neuropathy at the wrist as an early manifestation of diabetic neuropathy, Horinouchi 709–713
men and premenopausal women with type 2 diabetes
fracture is additionally attributed to hyperhomocysteinemia in men and premenopausal women with type 2 diabetes, Li 236–241
metabolic syndrome
is persistence of metabolic syndrome associated with poor health-related quality of life in non-diabetic Iranian adults? Tehran Lipid and Glucose Study, Amiri 687–693
metformin
effect of combination therapy with repaglinide and metformin hydrochloride on glycemic control in Japanese patients with type 2 diabetes mellitus, Kawamori 72–79
pioglitazone and metformin are equally effective in reduction of chemerin in patients with type 2 diabetes, Esteghamati 327–332
microinflammation
effect of renal impairment on cognitive function during a 3-year follow up in elderly patients with type 2 diabetes: association with microinflammation, Kawamura 597–605
midline-estimating statistic of rhythm
variations in 7-day/24-h circadian pattern of ambulatory blood pressure and heart rate of type 2 diabetes patients, Bhardwaj 728–733
monogenic diabetes
many faces of monogenic diabetes (Review Article), Schwitzgebe 121–133
mortality
clinical characteristics of hyperglycemic crises in patients without a history of diabetes, Chou 657–662
new-onset
clinical characteristics of hyperglycemic crises in patients without a history of diabetes, Chou 657–662
next generation sequencing
many faces of monogenic diabetes (Review Article), Schwitzgebe 121–133
non-alcoholic fatty liver disease
prevalences of diabetic retinopathy and nephropathy are lower in Korean type 2 diabetic patients with non-alcoholic fatty liver disease, Kim 170–175
novel antidiabetic agents
renal sodium glucose cotransporter 2 inhibitors as a novel therapeutic approach to treatment of type 2 diabetes: clinical data and mechanism of action (Review Article), Fujita 265–275
obesity
obesity as an effect modifier of the association between leptin and diabetic kidney disease, Hanai 213–220
decreased serum CA19-9 is associated with improvement of insulin resistance and metabolic control in patients with obesity and type 2 diabetes after Roux-en-Y gastric bypass, Tu 694–700
older diabetes patients
appropriate physical activity and dietary intake achieve optimal metabolic control in older type 2 diabetes patients, Huang 418–427
oral antidiabetic drugs
randomized, placebo-controlled, double-blind glycemic control trial of novel sodium-dependent glucose cotransporter 2 inhibitor ipragliflozin in Japanese patients with type 2 diabetes mellitus, Kashiwagi 382–391
changes in oral antidiabetic prescriptions and improved glycemic control during the years 2002–2011 in Japan (JDDM32), Oishi 581–587
oral glucose tolerance test
correlation between mean platelet volume and blood glucose levels after oral glucose loading in normoglycemic and prediabetic Japanese subjects, Shimodaira 66–71
accurate method to estimate insulin resistance from multiple regression models using data of metabolic syndrome and oral glucose tolerance test, Wu 290–296
oral glucose-stimulated serum C-peptide predicts successful switching from insulin therapy to liraglutide monotherapy in Japanese patients with type 2 diabetes and renal impairment, Araki 435–441
oral glucose tolerance test-based index
oral glucose tolerance test-based calculation identifies different glucose intolerance phenotypes within the impaired fasting glucose range, Schianca 533–538
oxidative stress
diabetic cardiomyopathy and its mechanisms: role of oxidative stress and damage (Review Article), Liu 623–634
pancreatic β-cells
palmitate induces reactive oxygen species production and β-cell dysfunction by activating nicotinamide adenine dinucleotide phosphate oxidase through Src signaling, Sato 19–26
postprandial C-peptide to glucose ratio as a predictor of β-cell function and its usefulness for staged management of type 2 diabetes, Lee 517–524
pancreatic transcription factors
*in vitro* quantitative and relative gene expression analysis of pancreatic transcription factors Pdx-1, Ngn-3, Isl-1, Pax-4, Pax-6 and Nkx-6.1 in trans-differentiated human hepatic progenitors, Vishwakarma 492–500
parasympathetic
comparative study on cardiac autonomic modulation during deep breathing test and diaphragmatic breathing in type 2 diabetes and healthy subjects, Subbalakshmi 456–463
persistency
is persistence of metabolic syndrome associated with poor health-related quality of life in non-diabetic Iranian adults? Tehran Lipid and Glucose Study, Amiri 687–693
personalized therapy
one size does not fit all glycemic targets for type 2 diabetes (Review Article), Pozzilli 134–141
peroxisome proliferator-activated receptor-gamma agonist
combination of a peroxisome proliferator-activated receptor-gamma agonist and an angiotensin II receptor blocker attenuates myocardial fibrosis and dysfunction in type 2 diabetic rats, Shim 362–371
peripheral nervous system
sensory and motor physiological functions are impaired in gastric inhibitory polypeptide receptor-deficient mice, Okawa 31–37
peroxisome proliferator activated receptor gamma gene
Role of peroxisome proliferator-activated receptor gamma gene polymorphisms in type 2 diabetes mellitus patients of West Bengal, India, Pattanayak 188–191
physical activity
appropriate physical activity and dietary intake achieve optimal metabolic control in older type 2 diabetes patients, Huang 418–427
pioglitazone
pioglitazone and metformin are equally effective in reduction of chemerin in patients with type 2 diabetes, Esteghamati 327–332
post-challenge plasma glucose
relationship and factors responsible for regulating fasting and post-challenge plasma glucose levels in the early stage development of type 2 diabetes mellitus, Aoyama-Sasabe 663–670
prediabetes
correlation between mean platelet volume and blood glucose levels after oral glucose loading in normoglycemic and prediabetic Japanese subjects, Shimodaira 66–71
potential association between prediabetic conditions and gingival and/or periodontal inflammation, Andriankaja 108–114
trends in the prevalence of type 2 diabetes and prediabetes in community-dwelling Japanese subjects: the Hisayama Study, Mukai 162–169
predictive factors
predictive factors of durability to sitagliptin: slower reduction of glycated hemoglobin, older age and higher baseline glycated hemoglobin, Chung 51–59
prevalence
trends in the prevalence of type 2 diabetes and prediabetes in community-dwelling Japanese subjects: the Hisayama Study, Mukai 162–169
prevalence and risk factors of development of peripheral diabetic neuropathy in type 2 diabetes mellitus in a tertiary care setting, Bansal 714–721
prevention
epidemic trend of diabetes in China (Review Article), Chen 478–481
productive age
prevalence and clinical profile of diabetes mellitus in productive aged urban Indonesians, Mihardja 507–512
propensity score-matched analysis
evaluation of insulin regimens as an effective option for glycemic control in patients with type 2 diabetes: a propensity score-matched cohort study across Japan (JDDM31), Kanatsuka 539–547
prospective study
Predicting type 2 diabetes using Sasang constitutional medicine, Cho 525–532
protein
interactive effects of an isocaloric high-protein diet and resistance exercise on body composition, ghrelin, and metabolic and hormonal parameters in untrained young men: a randomized clinical trial, Kim 242–247
quality of life
application of a health-related quality of life conceptual model in community-dwelling older Chinese people with diabetes to understand the relationships among clinical and psychological outcomes, Shiu 677–686
is persistence of metabolic syndrome associated with poor health-related quality of life in non-diabetic Iranian adults? Tehran Lipid and Glucose Study, Amiri 687–693
reactive oxygen species
palmitate induces reactive oxygen species production and β-cell dysfunction by activating nicotinamide adenine dinucleotide phosphate oxidase through Src signaling, Sato 19–26
relative quantification
*in vitro* quantitative and relative gene expression analysis of pancreatic transcription factors Pdx-1, Ngn-3, Isl-1, Pax-4, Pax-6 and Nkx-6.1 in trans-differentiated human hepatic progenitors, Vishwakarma 492–500
renal glucose reabsorption
renal sodium glucose cotransporter 2 inhibitors as a novel therapeutic approach to treatment of type 2 diabetes: clinical data and mechanism of action (Review Article), Fujita 265–275
renal impairment
oral glucose-stimulated serum C-peptide predicts successful switching from insulin therapy to liraglutide monotherapy in Japanese patients with type 2 diabetes and renal impairment, Araki 435–441
effect of renal impairment on cognitive function during a 3-year follow up in elderly patients with type 2 diabetes: association with microinflammation, Kawamura 597–605
repaglinide
effect of combination therapy with repaglinide and metformin hydrochloride on glycemic control in Japanese patients with type 2 diabetes mellitus, Kawamori 72–79
resistance exercise
interactive effects of an isocaloric high-protein diet and resistance exercise on body composition, ghrelin, and metabolic and hormonal parameters in untrained young men: a randomized clinical trial, Kim 242–247
retinol-binding protein 4 gene and protein expression
effect of endurance training on retinol-binding protein 4 gene expression and its protein level in adipose tissue and the liver in diabetic rats induced by a high-fat diet and streptozotocin, Mansouri 484–491
reward-based
reward-based, task-setting education strategy on glycemic control and self-management for low-income outpatients with type 2 diabetes, Guo 410–417
risk
comparison of four cardiovascular risk prediction functions among Chinese patients with diabetes mellitus in the primary care setting, Jiao 606–614
risk factors
prevalence and risk factors of development of peripheral diabetic neuropathy in type 2 diabetes mellitus in a tertiary care setting, Bansal 714–721
Sasang constitutional medicine
predicting type 2 diabetes using Sasang constitutional medicine, Cho 525–532
self medication
one-min mental status examination for category fluency is more useful than mini-mental state examination to evaluate the reliability of insulin self-injection in elderly diabetic patients, Yajima 340–344
serine racemase gene
association of serine racemase gene variants with type 2 diabetes in the Chinese Han population, Zhang 286–289
shear stress
combined effects of physiologically relevant disturbed wall shear stress and glycated albumin on endothelial cell functions associated with inflammation, thrombosis and cytoskeletal dynamics, Maria 372–381
single-nucleotide polymorphisms
association of serine racemase gene variants with type 2 diabetes in the Chinese Han population, Zhang 286–289
sitagliptin
predictive factors of durability to sitagliptin: slower reduction of glycated hemoglobin, older age and higher baseline glycated hemoglobin, Chung 51–59
efficacy and safety of sitagliptin in Japanese patients with type 2 diabetes switched from glinides, Tanaka 199–205
sitagliptin improves albuminuria in patients with type 2 diabetes mellitus, Mori 313–319
comparison of the efficacy of sitagliptin and glimepiride dose-up in Japanese patients with type 2 diabetes poorly controlled by sitagliptin and glimepiride in combination, Shimoda 320–326
factors influencing the durability of the glucose-lowering effect of sitagliptin combined with a sulfonylurea, Kubota 445–448
short- and long-term effect of sitagliptin after near normalization of glycemic control with insulin in poorly controlled Japanese type 2 diabetic patients, Fujisawa 548–553
sodium-dependent glucose co-transporter 2
Randomized, placebo-controlled, double-blind glycemic control trial of novel sodium-dependent glucose cotransporter 2 inhibitor ipragliflozin in Japanese patients with type 2 diabetes mellitus, Kashiwagi 382–391
sodium glucose cotransporter 2 inhibitors
renal sodium glucose cotransporter 2 inhibitors as a novel therapeutic approach to treatment of type 2 diabetes: clinical data and mechanism of action (Review Article), Fujita 265–275
sphingosine-1-phosphate
modulation of sphingosine-1-phosphate and apolipoprotein M levels in the plasma, liver and kidneys in streptozotocin-induced diabetic mice, Nojiri 639–648
steady-state plasma glucose
accurate method to estimate insulin resistance from multiple regression models using data of metabolic syndrome and oral glucose tolerance test, Wu 290–296
stem cells
number of circulating pro-angiogenic cells, growth factor and anti-oxidative gene profiles might be altered in type 2 diabetes with and without diabetic foot syndrome, Nowak 99–107
streptozotocin
modeling type 2 diabetes in rats using high fat diet and streptozotocin (Review Article), Skovsø 349–358
streptozotocin-induced diabetes mice
modulation of sphingosine-1-phosphate and apolipoprotein M levels in the plasma, liver and kidneys in streptozotocin-induced diabetic mice, Nojiri 639–648
structural equation modeling
application of a health-related quality of life conceptual model in community-dwelling older Chinese people with diabetes to understand the relationships among clinical and psychological outcomes, Shiu 677–686
sudomotor function
correlation between sudomotor function, sweat gland duct size and corneal nerve fiber pathology in patients with type 2 diabetes mellitus, Ishibashi 588–596
sulfonylurea
factors influencing the durability of the glucose-lowering effect of sitagliptin combined with a sulfonylurea, Kubota 445–448
sweat gland duct
correlation between sudomotor function, sweat gland duct size and corneal nerve fiber pathology in patients with type 2 diabetes mellitus, Ishibashi 588–596
task-setting
reward-based, task-setting education strategy on glycemic control and self-management for low-income outpatients with type 2 diabetes, Guo 410–417
total insulin dose
basal insulin requirement of youth with type 1 diabetes differs according to age, Urakami 442–444
trans-differentiation
*in vitro* quantitative and relative gene expression analysis of pancreatic transcription factors Pdx-1, Ngn-3, Isl-1, Pax-4, Pax-6 and Nkx-6.1 in trans-differentiated human hepatic progenitors, Vishwakarma 492–500
type 1 diabetes
diagnostic criteria for acute-onset type 1 diabetes mellitus (2012): report of the Committee of Japan Diabetes Society on the Research of Fulminant and Acute-onset Type 1 Diabetes Mellitus, Kawasaki 115–118
trends in the prevalence of type 2 diabetes and prediabetes in community-dwelling Japanese subjects: the Hisayama Study, Mukai 162–169
basal insulin requirement of youth with type 1 diabetes differs according to age, Urakami 442–444
type 1 diabetes mellitus
diurnal variation of carbohydrate insulin ratio in adult type 1 diabetic patients treated with continuous subcutaneous insulin infusion, Nakamura 48–50
identification of novel risk genes associated with type 1 diabetes mellitus using a genome-wide
gene-based association analysis, Qiu 649–656
type 2 diabetes
efficacy and safety of sitagliptin in Japanese patients with type 2 diabetes switched from glinides, Tanaka 199–205
modeling type 2 diabetes in rats using high fat diet and streptozotocin (Review Article), Skovsø 349–358
low high-density lipoprotein cholesterol level is a significant risk factor for development of type 2 diabetes: data from the Hawaii–Los Angeles–Hiroshima study, Hirano 501–506
tolerability, effectiveness and predictive parameters for the therapeutic usefulness of exenatide in obese, Korean patients with type 2 diabetes, Song 554–562
secretory units of islets in transplantation index is a useful predictor of insulin requirement in Japanese type 2 diabetic patients, Iwata 570–580
correlation between sudomotor function, sweat gland duct size and corneal nerve fiber pathology in patients with type 2 diabetes mellitus, Ishibashi 588–596
relationship and factors responsible for regulating fasting and post-challenge plasma glucose levels in the early stage development of type 2 diabetes mellitus, Aoyama-Sasabe 663–670
mild-to-moderate intensity exercise improves cardiac autonomic drive in type 2 diabetes, Goit 722–727
type 2 diabetes mellitus
dietary intake in Japanese patients with type 2 diabetes: analysis from Japan Diabetes Complications Study, Horikawa 176–187
role of peroxisome proliferator-activated receptor gamma gene polymorphisms in type 2 diabetes mellitus patients of West Bengal, India, Pattanayak 188–191
comparison of three α-glucosidase inhibitors for glycemic control and bodyweight reduction in Japanese patients with obese type 2 diabetes, Sugihara 206–212
association of serine racemase gene variants with type 2 diabetes in the Chinese Han population, Zhang 286–289
postprandial C-peptide to glucose ratio as a predictor of β-cell function and its usefulness for staged management of type 2 diabetes, Lee 517–524
predicting type 2 diabetes using Sasang constitutional medicine, Cho 525–532
evaluation of insulin regimens as an effective option for glycemic control in patients with type 2 diabetes: a propensity score-matched cohort study across Japan (JDDM31), Kanatsuka 539–547
changes in oral antidiabetic prescriptions and improved glycemic control during the years 2002–2011 in Japan (JDDM32), Oishi 581–587
efficacy of glimepiride/metformin fixed-dose combination vs metformin uptitration in type 2 diabetic patients inadequately controlled on low-dose metformin monotherapy: a randomized, open label, parallel group, multicenter study in Korea, Kim 701–708
urban Indonesians
prevalence and clinical profile of diabetes mellitus in productive aged urban Indonesians, Mihardja 507–512
vibratory sensation
assessment of vibratory sensation with a tuning fork at different sites in Japanese patients with diabetes mellitus, Takahara 90–93
visceral fat
effects of bariatric surgery on metabolic and nutritional parameters in severely obese Korean patients with type 2 diabetes: a prospective 2-year follow up, Kim 221–227
well-being
weight loss on a structured hypocaloric diet with or without exercise improves emotional distress and quality of life in overweight and obese patients with type 2 diabetes, Wycherley 94–98
Westernized lifestyle
low high-density lipoprotein cholesterol level is a significant risk factor for development of type 2 diabetes: data from the Hawaii–Los Angeles–Hiroshima study, Hirano 501–506
workers
increasing risk of diabetes mellitus according to liver function alterations in electronic workers, Lee 671–676
wound healing
macrophage stimulating agent soluble yeast β-1,3/1,6-glucan as a topical treatment of diabetic foot and leg ulcers: a randomized, double blind, placebo-controlled phase II study, Zykova 392–399
youth
comparison of brachial artery flow-mediated dilation in youth with type 1 and type 2 diabetes mellitus, Ohsugi 615–620

